# Supplementary material for: Conjugation with Phospholipids as a Modification Increasing Anticancer Activity of Phenolic Acids in Metastatic Melanoma—In Vitro and In Silico Studies
Source: Int J Mol Sci. 2021 Aug 5;22(16):8397. doi: 10.3390/ijms22168397 (PMC8395125; doi:10.3390/ijms22168397)
Supplement: Supplementary file 1 [file ijms-22-08397-s001.zip › supplemantary figure legends.pdf]

Supplementary figures' legends:

Figure S1. Representative images of normal skin fibroblasts (NHDF cells) in the presence of selected compounds (at 100 μM).

Figure S2. The influence of ANISA and its conjugates (A) and VA and its conjugates (B) on the reactive oxygen species formation in NHDF cells. The results were presented as percentages of the control (no compound). In case of acids ANISA and VA, statistical significance was compared to the samples containing no compounds (# $p<0.05$; ## $p<0.001$) whereas in case of PC-conjugates to the samples containing ANISA (A) or VA (B) (* $p<0.05$; ** $p<0.001$).

Figure S3. Progression of NHDF cell cycle in the presence of the studied compounds. Cells were cultured for 72 h in the presence of the studied chemicals (at 100 μM). The distribution of studied cells in major phases of the cycle is presented (A). Representative histograms for effects obtained in NHDF incubated with selected compounds were shown (B).

Figure S4. Induction of apoptosis in NHDF cells in the presence of the studied compounds. The cells were cultured for 72 h in the presence of the studied chemicals (at 100 μM). The percentage of necrotic, early and late apoptotic cells was determined (A). Representative histograms recorded for selected compounds were shown (B).
