# Supplementary figures and images for: Conjugation with Phospholipids as a Modification Increasing Anticancer Activity of Phenolic Acids in Metastatic Melanoma—In Vitro and In Silico Studies
Source: Int J Mol Sci. 2021 Aug 5;22(16):8397. doi: 10.3390/ijms22168397 (PMC8395125; doi:10.3390/ijms22168397)

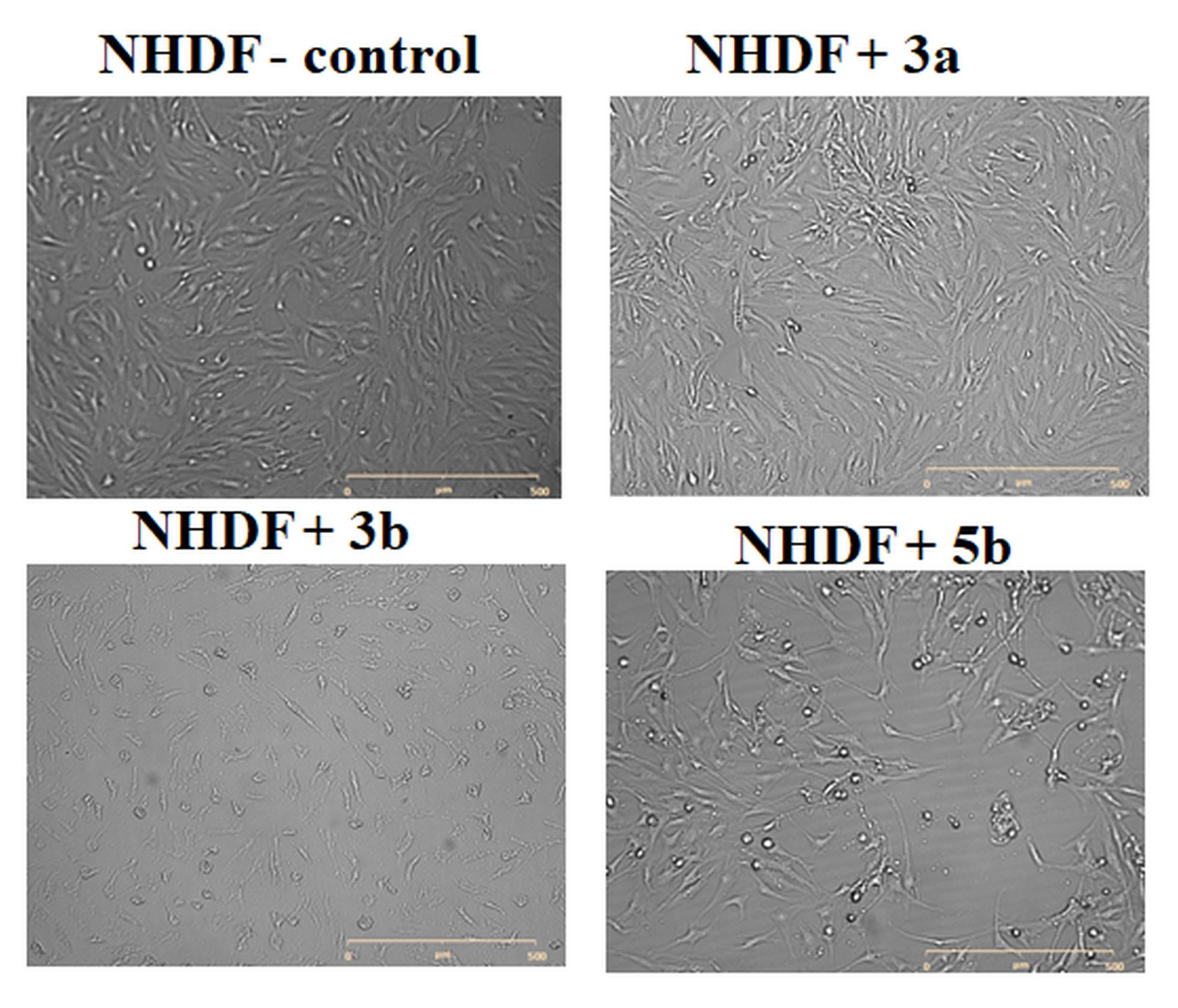

Supplement: Supplementary file 1 [file ijms-22-08397-s001.zip › fig s1.jpg]

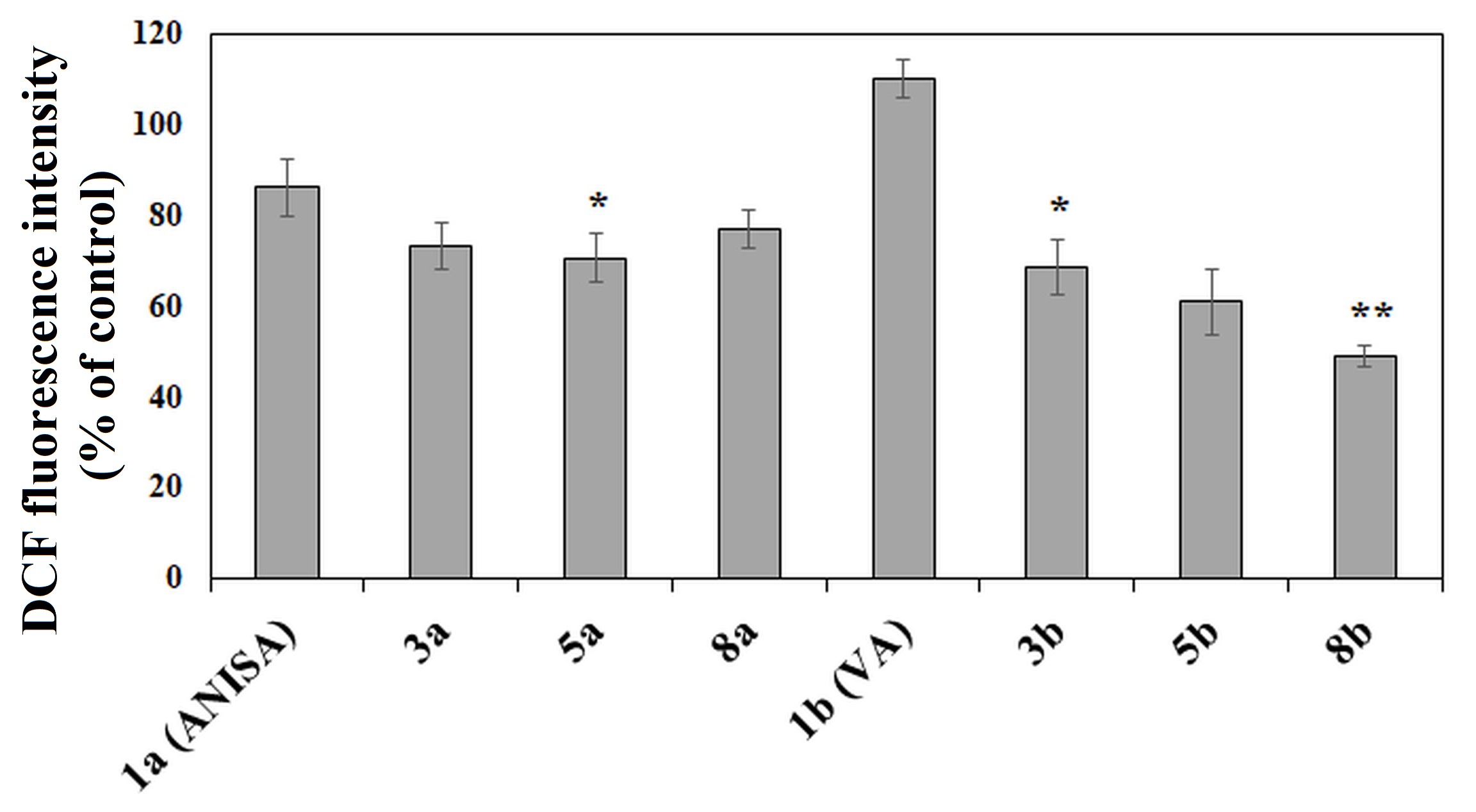

Supplement: Supplementary file 1 [file ijms-22-08397-s001.zip › fig s2.jpg]

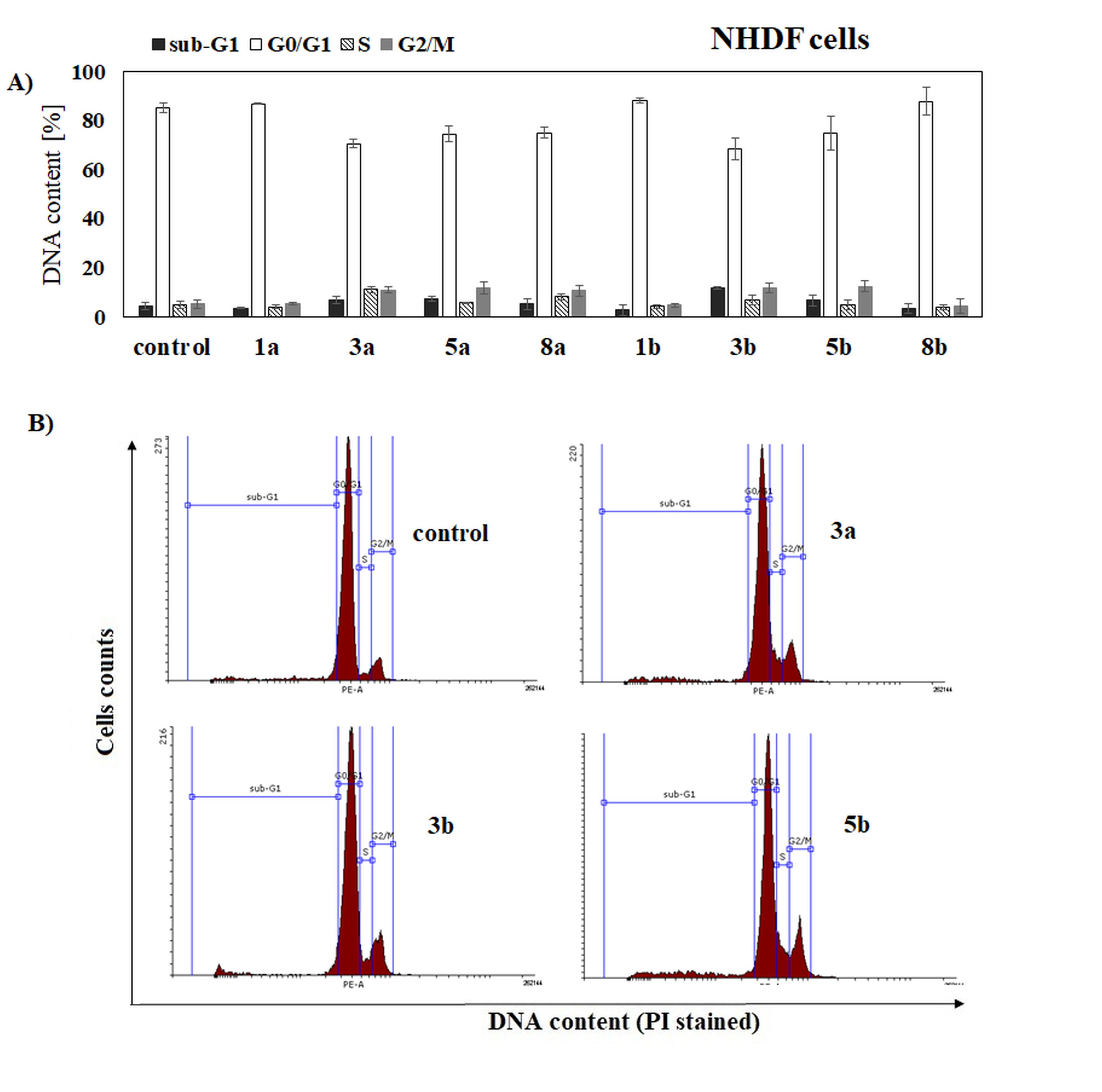

Supplement: Supplementary file 1 [file ijms-22-08397-s001.zip › fig s3.jpg]

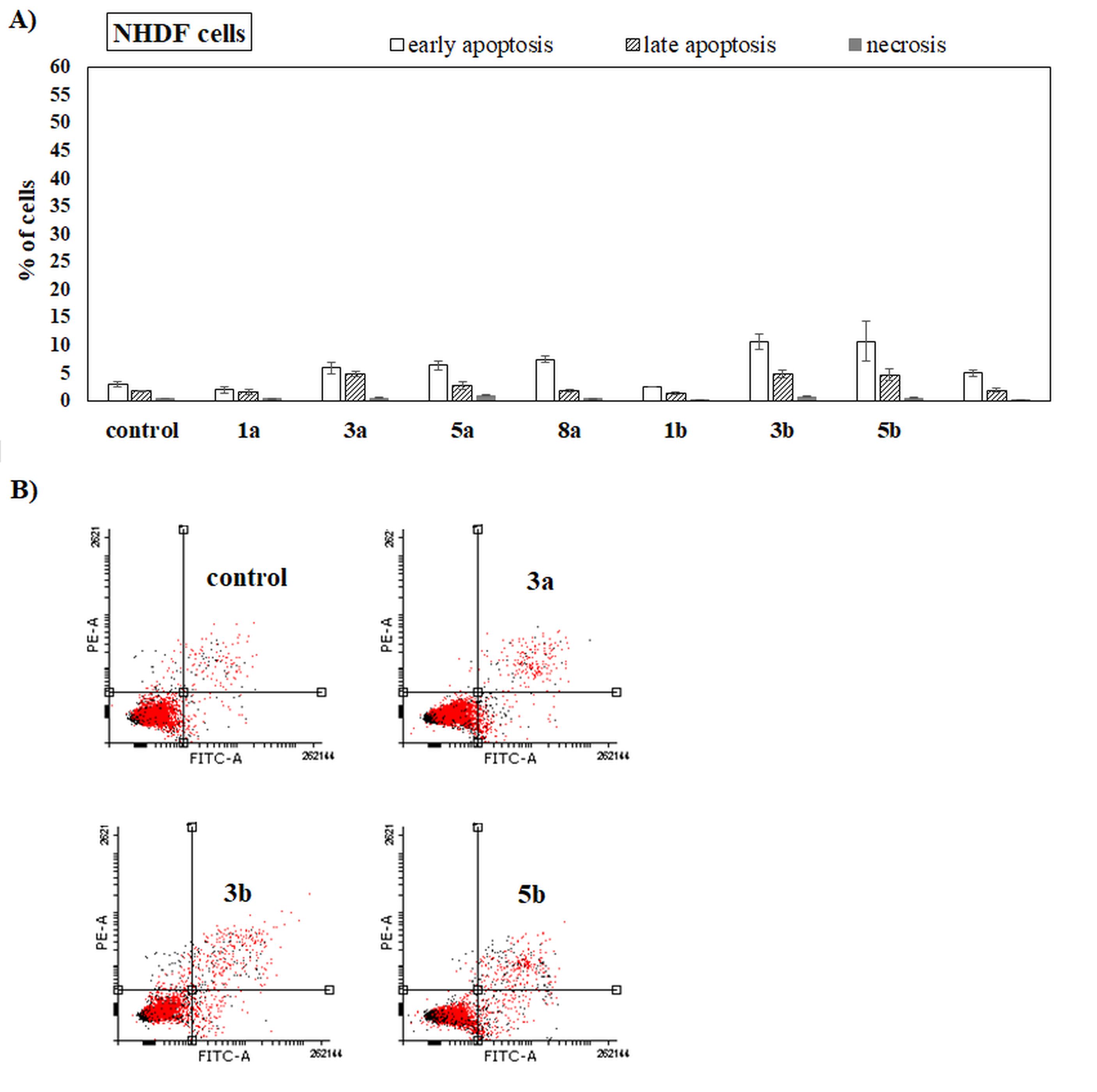

Supplement: Supplementary file 1 [file ijms-22-08397-s001.zip › fig s4.jpg]
